# The Antiviral RNAi Response in Vector and Non-vector Cells against Orthobunyaviruses

**DOI:** 10.1371/journal.pntd.0005272

**Published:** 2017-01-06

**Authors:** Isabelle Dietrich, Xiaohong Shi, Melanie McFarlane, Mick Watson, Anne-Lie Blomström, Jessica K. Skelton, Alain Kohl, Richard M. Elliott, Esther Schnettler

**Affiliations:** 1 MRC-University of Glasgow Centre for Virus Research, Glasgow, Scotland, United Kingdom; 2 Roslin Institute, Edinburgh, Scotland, United Kingdom; Tulane School of Public Health and Tropical Medicine, UNITED STATES

## Abstract

**Background:**

Vector arthropods control arbovirus replication and spread through antiviral innate immune responses including RNA interference (RNAi) pathways. Arbovirus infections have been shown to induce the exogenous small interfering RNA (siRNA) and Piwi-interacting RNA (piRNA) pathways, but direct antiviral activity by these host responses in mosquito cells has only been demonstrated against a limited number of positive-strand RNA arboviruses. For bunyaviruses in general, the relative contribution of small RNA pathways in antiviral defences is unknown.

**Methodology/Principal Findings:**

The genus *Orthobunyavirus* in the *Bunyaviridae* family harbours a diverse range of mosquito-, midge- and tick-borne arboviruses. We hypothesized that differences in the antiviral RNAi response in vector versus non-vector cells may exist and that could influence viral host range. Using *Aedes aegypti*-derived mosquito cells, mosquito-borne orthobunyaviruses and midge-borne orthobunyaviruses we showed that bunyavirus infection commonly induced the production of small RNAs and the effects of the small RNA pathways on individual viruses differ in specific vector-arbovirus interactions.

**Conclusions/Significance:**

These findings have important implications for our understanding of antiviral RNAi pathways and orthobunyavirus-vector interactions and tropism.

## Author Summary

A number of orthobunyaviruses such as Oropouche virus, La Crosse virus and Schmallenberg virus are important global human or animal pathogens transmitted by arthropod vectors. Further understanding of the antiviral control mechanisms in arthropod vectors is key to developing novel prevention strategies based on preventing transmission. Antiviral small RNA pathways such as the exogenous siRNA and piRNA pathways have been shown to mediate antiviral activity against positive-strand RNA arboviruses, but information about their activities against negative-strand RNA arboviruses is critically lacking. Here we show that in *Aedes aegypti*-derived mosquito cells, the antiviral responses to mosquito-borne orthobunyaviruses is largely mediated by both siRNA and piRNA pathways, whereas the piRNA pathway plays only a minor role in controlling midge-borne orthobunyaviruses. This suggests that vector specificity is in part controlled by antiviral responses that depend on the host species. These findings contribute significantly to our understanding of arbovirus-vector interactions.

## Introduction

Orthobunyaviruses are endemic in tropical and subtropical regions worldwide and are transmitted by mosquitoes, midges, ticks or other arthropods. The *Orthobunyavirus* genus within the *Bunyaviridae* family comprises at least 30 viruses that can cause disease in humans, including Oropouche virus (OROV; febrile illness), La Crosse virus (LACV; encephalitis) and Ngari virus (haemorrhagic fever) [1]. In addition, infection by orthobunyaviruses such as Cache Valley virus (CVV) and Schmallenberg virus (SBV) can lead to disease in animals [2].

Bunyamwera virus (BUNV) is the prototype virus of both the *Orthobunyavirus* genus and the family. Like most viruses in the genus, the BUNV genome possesses a tripartite, single-stranded negative sense RNA genome, in which the small (S) segment encodes the nucleocapsid (N) protein and the nonstructural protein NSs in overlapping reading frames, the medium (M) segment encodes a viral glycoprotein precursor (in the order Gn-NSm-Gc) for two envelope glycoproteins Gn, Gc and a nonstructural protein NSm, and the large (L) segment encodes the RNA-dependent RNA polymerase. This genome structure is generally reflected by most orthobunyaviruses with some differences for example in the presence or length of NSs [1].

BUNV was originally isolated from *Aedes* spec. mosquitoes in the Semliki Forest in Uganda in 1943 [3] but has since also been found in *Culex* spec. and *Mansonia* spec. (see [4] and references on BUNV therein) and *Ochlerotatus* spec. [5]. BUNV infections cause febrile illness and (rarely) encephalitis in humans in Sub-Saharan Africa, in particular in Nigeria and the Central African Republic, with wild rodents likely to be serving as amplifying reservoir [6]. Cache Valley virus (CVV) belongs to the Bunyamwera serogroup and is enzootic throughout North and South America [7, 8]. It was first isolated from *Culiseta inornata* mosquitoes in the Cache Valley in Utah, United States of America in 1956 [8], and has since been shown to be transmitted by mosquitoes of the *Culiseta*, *Anopheles*, *Aedes*, *Culex* and *Ochleratatus* genera [9, 10]. A small number of CVV infections in humans have been reported [11–13], where infection rarely leads to serious disease. In ruminants, including sheep and cattle, CVV causes spontaneous abortions and multiple congenital malformations [14–16]. Large mammals including deer, horses and sheep are known to serve as amplifying hosts [6].

In addition to mosquito-borne orthobunyaviruses some members of the genus are exclusively transmitted by biting midges (e.g. SBV, OROV and Sathuperi virus [SATV]) [17–22], ticks (Tete group viruses Bahig and Matruh) [23] or are mosquito/insect-specific [24, 25].

SBV and SATV are closely related orthobunyaviruses of the Simbu serogroup. SBV was first discovered in 2011 in Germany and the Netherlands [26] and infections have since been reported in many European countries [27]. Infections can lead to reduced milk yield, fever, fetal malformations and abortions in ruminants (primarily sheep, goats and cattle) [26, 28]; human infections have not been reported. SATV was isolated from mosquitoes in India in 1957 [29], and was later detected in cattle and biting midges in Nigeria [30, 31]. More recently, SATV was detected in Japan in 1999 [32]. To date little information is available on its pathogenicity in ruminants [22]. Both SBV and SATV are transmitted by *Culicoides* sp. biting midges [22, 31, 33–35].

The exogenous small interfering (exo-si) RNA and Piwi-interacting (pi)RNA pathways have previously been described as important mosquito antiviral responses limiting the replication of positive-strand RNA flaviviruses and togaviruses [36, 37]. The activity of the exo-siRNA pathway is mediated by two key proteins, the endoribonuclease Dicer-2 (Dcr2) and Argonaute-2 (Ago2). Dcr2 cleaves long virus-derived double-stranded RNAs (dsRNAs) into 21 nucleotides (nt) long small interfering RNA (viRNA) duplexes. These viRNAs are then incorporated into the multiprotein RNA-induced silencing complex (RISC), where presumably one strand is retained (guide strand) by Ago2 to detect, bind and catalyze the degradation of complementary single-stranded (ss)RNA such as viral mRNA. Indeed, 21 nt viRNAs were produced in mosquitoes or mosquito-derived cell lines upon infection with several arboviruses, for example flaviviruses (dengue virus, DENV; West Nile virus, WNV), alphaviruses of the *Togaviridae* family (chikungunya virus, CHIKV; Semliki Forest virus, SFV; Sindbis virus, SINV; o’nyong’nyong virus, ONNV), bunyaviruses (Rift Valley fever virus, RVFV; and SBV) as well as reoviruses (bluetongue virus, BTV) [38–42]. Silencing experiments in mosquitoes have confirmed the antiviral activity of the exo-siRNA pathway against DENV, SINV, CHIKV and ONNV *in vivo* [42–46].

In addition to the exo-siRNA pathway, the piRNA pathway has been shown to be a contributor to antiviral immunity in mosquitoes or derived cells [38, 47]. In *Drosophila*, the piRNA pathway is involved in the epigenetic regulation of the expression of transposable elements (TE) in the germline, and thus preserves the genome integrity [48–51]. However, PIWI proteins have also been detected in somatic cells [49, 52–54]. The production of transposon-specific piRNAs is complex and relies on proteins of PIWI clade, in particular Piwi, Aubergine (Aub) and Argonaute-3 (Ago3). piRNAs are believed to be generated via a primary processing pathway and a secondary ping-pong amplification loop [51, 52]. Primary piRNAs have a 5’ uridine (U_1_) bias. Secondary piRNAs have a 10 nt overlap with primary piRNAs and contain an adenine at position 10 (A_10_ bias). Mature piRNAs are generally 26–32 nt in length. The piRNA pathway is functional in mosquito germline and somatic cells [38, 39, 47, 55–57]. Interestingly, in mosquitoes a loss of Aub and a diversification of Piwi proteins has occurred [58]. This gene diversification has been linked with a gain of function of the piRNA pathway and a new role in antiviral immunity since the pathway has also been found to target a number of mosquito-borne viruses including DENV, CHIKV, SFV and RVFV [38, 39, 47]. Further, in the *Ae*. *aegypti*-derived Aag2 cell line an antiviral effect of Piwi4 against SFV has been directly demonstrated [47]. Recently it was shown that Ago3 and Piwi5 (and Piwi6 to a lesser extent) are needed for the generation of SINV and DENV specific piRNAs in Aag2 cells [59, 60]; however, the viral RNA substrate that induces this pathway is unknown. Importantly, the respective contribution of the two small RNA pathways to immune defenses against negative-sense RNA arboviruses has not been studied. In short, little is known about the interactions of viruses and vectors, and antiviral responses of vectors, which may govern viral infection, dissemination and transmission.

In this study we investigated the antiviral activities of the mosquito exo-siRNA and piRNA pathway against two mosquito- and three midge-borne orthobunyaviruses. Using reporter BUNV and SBV that express Nano luciferase we compared these responses in vector-virus and non-vector-virus interactions. We performed small RNA sequencing and showed that mosquito as well as midge-borne viruses produce virus-specific siRNAs and piRNAs in mosquito cells. Interestingly we found that silencing of Ago2 and Piwi4 in Aag2 cells led to increased viral replication of mosquito-borne orthobunyaviruses, in contrast to midge-borne orthobunyaviruses where only Ago2 silencing increased virus replication. Additionally, silencing of other piRNA pathway members (Piwi5, 6 and Ago3) affected virus replication differently depending on whether the virus was mosquito- or midge borne. These findings indicate that RNAi pathways play a crucial role in the control of orthobunyavirus replication; however, the piRNA pathway in Aag2 cells seems to be adapted to specific virus-vector combinations and may have important consequences for arbovirus tropism and vector specificity.

## Results

### Induction of small RNA production by orthobunyaviruses in mosquito and midge cells

**Fig 1:**
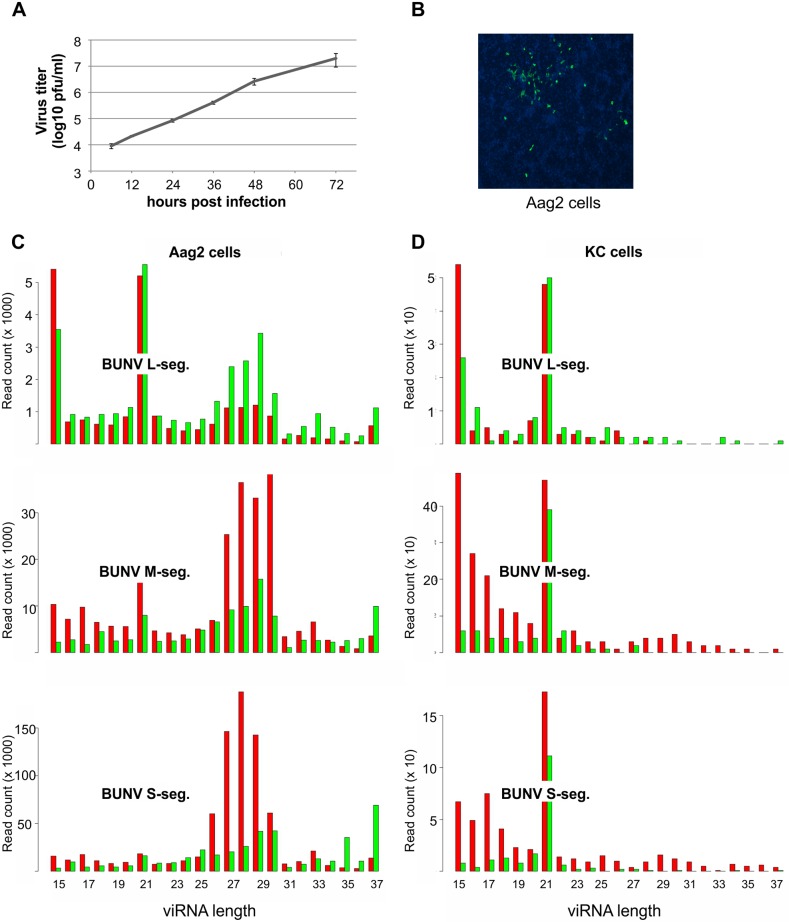
BUNV growth and BUNV-specific viRNA production in Aag2 cells. **(A)** BUNV growth curve following infection of Aag2 cells at MOI 1, determined by plaque assay. **(B)** Immunofluorescence of BUNV-infected Aag2 cells (MOI 0.01) at 48 hours p.i., using BUNV-N specific primary antibody and Alexa 488-conjugated anti-rabbit-IgG secondary antibody (green). Nucleus stained by Dapi (blue). **(C)** BUNV-specific small RNAs produced in infected Aag2 cells at 24 hours p.i. against L, M and S segments. The y axis indicates read frequency; the x axis indicates the length of the small RNAs (nt). Red indicates the small RNAs mapping to the antigenome and green the small RNAs mapping to the genome. **(D)** BUNV-specific small RNAs produced in infected KC cells at 48 hours p.i. against L, M and S segments. The y axis indicates read frequency; the x axis indicates the length of the small RNAs. Red indicates the small RNAs mapping to the antigenome and green the small RNAs mapping to the genome.

**Fig 2:**
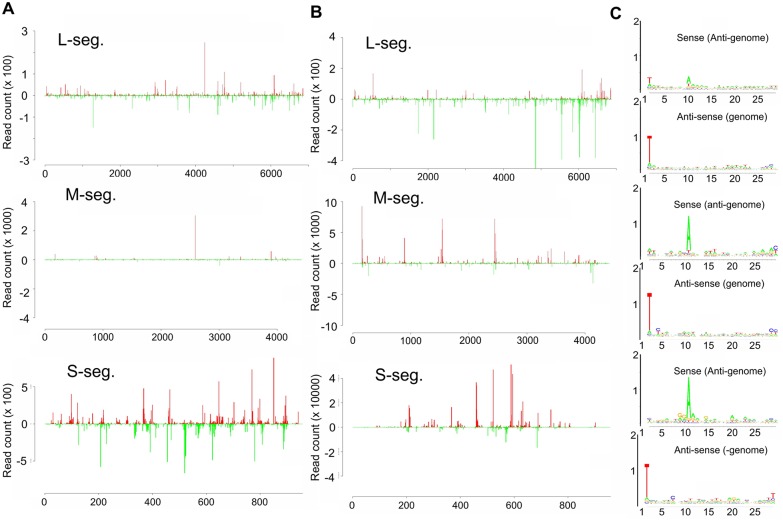
BUNV-specific small RNA and piRNA-like molecules produced in infected Aag2 cells at 24 hours p.i. **(A)** Frequency distribution of 21 nt small RNAs along the BUNV antigenome from the 5` to 3` (positive numbers and red), genome (negative numbers and green) from 3` to 5`. **(B)** Frequency distribution of 24–30 nt small RNAs along the BUNV antigenome from the 5` to 3` (positive numbers and red), genome (negative numbers and green) from 3` to 5` **(C)** Relative nucleotide frequency and conservation per position of 24–30 nt small RNAs mapping to the genome or antigenome of BUNV S, M and L segments. Sequence is represented as DNA.

Our previous work has shown that small RNAs with viRNA and piRNA characteristics are produced in non-vector mosquito cells infected with the midge-borne SBV [40] (S1A Fig). To determine if this is similar for a mosquito-borne virus, *Ae*. *aegypti*-derived Aag2 cells were infected with BUNV (Fig 1A and 1B) and small RNAs were isolated, sequenced and mapped to the virus genome and antigenome. As shown in Figs 1C and 2A, 21 nt long small RNAs were produced from all three segments which mapped along the genome and antigenome in a cold and hot spot pattern. For the L segment, these 21 nts viRNAs were the major small RNA species produced. Moreover, small RNAs of 24–30 nts with piRNA-specific features (A_10_, U_1_ bias), which mapped across the genome and antigenome in a hot and cold spot pattern (Fig 2B), were produced for all three segments (Fig 2C); however, they were the major small RNA species only for M and S segments with a bias for small RNAs mapping to the antigenome. In contrast, 24–30 nt small RNAs mapping to the L segment had a bias for the genome (Fig 1C). Similar results were obtained in BUNV-infected *Ae*. *albopictus*-derived U4.4 cells (S2 Fig).

To determine if similar small RNAs are produced for BUNV and SBV in midge cells; experiments were repeated with infected *C*. *sonorensis* KC cells. As previously reported no piRNA-like molecules could be detected for SBV in KC cells, in contrast, 21 nt small RNAs were mapped to the genome and antigenome of all three segments [40] (S1B Fig). Similar results were observed for BUNV small RNAs in infected KC cells (Fig 1D). Although small RNAs of 24–28 nts could be detected in KC cells, they lacked the piRNA-specific features (A_10_, U_1_ bias and 10 nts overlap of sense and antisense small RNAs) (Fig 1D).

Overall, our data showed that BUNV infection induced small RNA patterns comparable with SBV [40] in mosquito-derived cells as well as *Culicoides*-derived cells. In addition, differences in small RNA patterns were found between mosquito and midge cells.

### Generation of BUNV reporter virus expressing Nano luciferase

Previously, luciferase expressing alphaviruses were employed to investigate the antiviral RNAi response in arthropod cells [47, 61, 62]. To obtain similar tools for bunyaviruses, Nano luciferase (NL) expressing BUNV (BUNV-NL) and SBV (SBV-NL) were constructed in the course of this study. BUNV NSm protein, which is dispensable in viral replication in tissue culture, consists of the ectodomain, transmembrane, cytoplasmic domain and type-II transmembrane domain that also serves as internal Gc signal peptide [63, 64]. For BUNV-NL, the 62 residues of the NSm cytoplasmic domain (residues 395 to 455) was replaced by the NL coding region between residues 394 and 456, resulting in a chimeric NSm-NL fusion protein (S3A Fig). NSm-NL has a similar molecular weight to that of N protein (28.67 versus 26.67 kDa) and was indistinguishable from the N protein band in the protein profile of the reporter virus (S3B Fig). BUNV-NL exhibited smaller plaque size than the wildtype virus (S3C Fig). The reporter virus could readily infect Aag2 cells, similar to wildtype virus (S3D Fig). The SBV-NL reporter virus was constructed in the same way as BUNV-NL and successful infection of Aag2 cells, comparable to wildtype SBV, was verified by immunostaining (S3D Fig).

### piRNA and miRNA pathways differentially affect midge- and mosquito-borne virus infections in Aag2 cells

**Fig 3:**
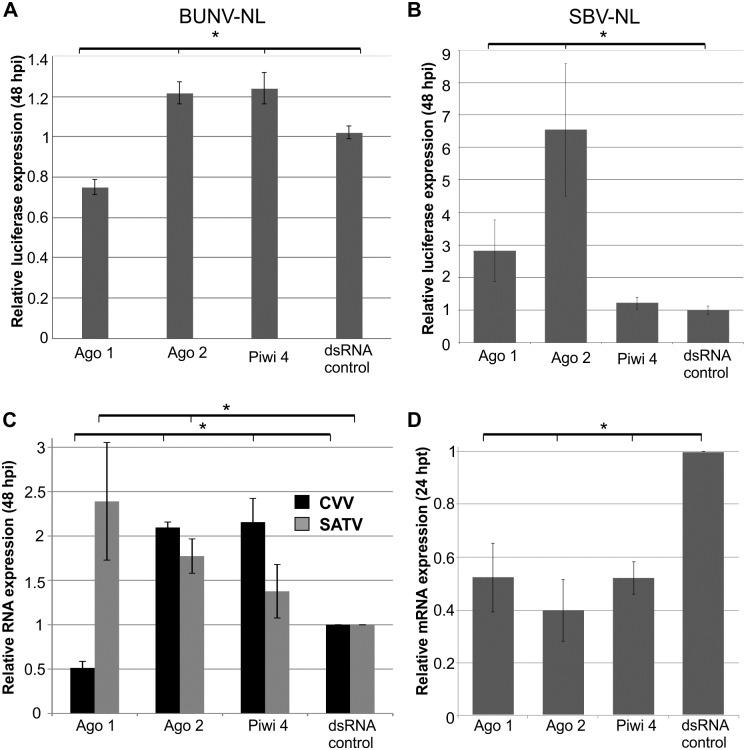
Effects of silencing exo-siRNA, miRNA and piRNA pathway key proteins on different orthobunyavirus infections in Aag2 cells. Silencing of key transcripts by transfection of sequence-specific dsRNA (Ago1, Ago2, Piwi4 or eGFP as negative control) into Aag2 cells, followed by infection at MOI 0.01 at 24 hours p.t.; BUNV-NL **(A)**, SBV-NL **(B)** or CVV, SATV **(C)**. Cells were lysed at 48 hours p.i. and luciferase activity determined **(A, B)** or RNA isolated, followed by cDNA synthesis and virus specific qRT-PCR **(C)**. Knockdown of RNAi transcripts was verified by qRT-PCR **(D)**. S7 was used as internal control in qRT-PCR assays. All data were normalized to cells transfected with control dsRNA. Graphs represent three independent experiments performed in triplicate for the luciferase assays and three independent experiments for the qRT-PCR assays. * p≤0.05 student t-test.

**Fig 4:**
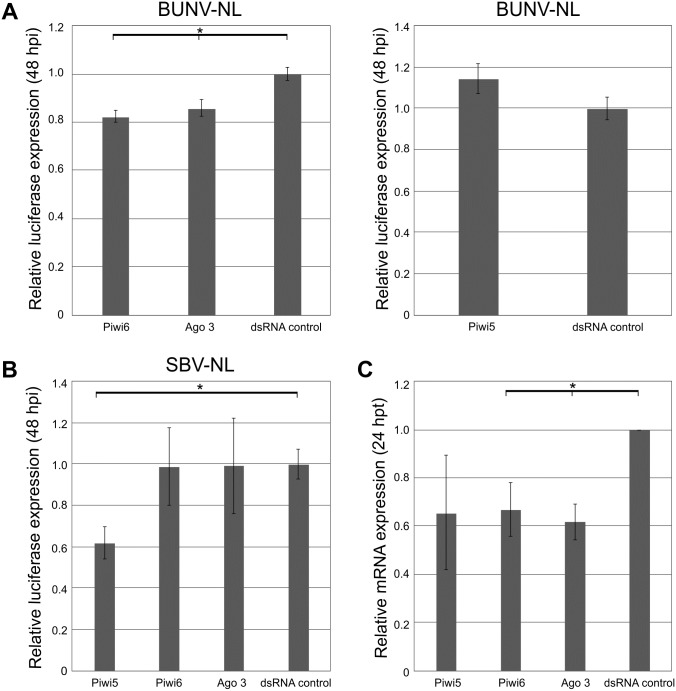
Effects of silencing Piwi5, Piwi6 and Ago3 on BUNV-NL and SBV-NL replication in Aag2 cells. Silencing of transcripts by transfection of sequence-specific dsRNA (Piwi5, Piwi6, Ago3 or eGFP as negative control) into Aag2 cells, followed by infection at MOI 0.01 at 24 hours p.t.; BUNV-NL **(A)**, SBV-NL **(B)**. Cells were lysed at 48 hours p.i. and luciferase activity determined **(A-B)**. Knockdown of RNAi transcripts was verified by qRT-PCR **(C)**. S7 was used as internal control in qRT-PCR assays. All data were normalized to cells transfected with control dsRNA. Graphs represent three independent experiments performed in triplicate for the luciferase assays and three independent experiments for the qRT-PCR assays. * p≤0.05 student t-test.

To assess the antiviral role of the small RNAi pathways in BUNV and SBV infected Aag2 mosquito cells, knockdown experiments were performed. Transcripts of the different RNAi pathway key effectors (Ago2, exo-siRNA pathway; Ago1, miRNA pathway; Piwi4-6 and Ago3, piRNA pathway) were silenced by transfection of sequence-specific dsRNA. The effect of the silencing was evaluated by BUNV-NL or SBV-NL infection at 24 hours post-transfection (p.t.) at low MOI (0.01) and subsequent luciferase detection at 48 hours p.i. (Figs 3A, 3B, 4A and 4B); dsRNA specific to eGFP was used as negative control.

Silencing of Ago2 led to an increase in luciferase expression for both BUNV-NL and SBV-NL (Fig 3A and 3B). In contrast, silencing of Piwi4 had no effect on SBV-NL, but resulted in an increase of BUNV-NL replication. Interestingly, silencing expression of other piRNA pathway related genes resulted in a significant decrease of BUNV-NL (Piwi6 and Ago3 knockdowns) and SBV-NL (Piwi5 knockdown) replication (Fig 4A and 4B). A decrease of luciferase expression was also observed for BUNV-NL following Ago1 silencing, compared to an increase of luciferase expression for SBV-NL (Fig 3A and 3B). These results suggested differences in the ability of the piRNA and miRNA pathway to interact with BUNV and SBV.

To determine if this was virus-specific or due to vector versus non-vector arbovirus interaction, silencing experiments were repeated with the mosquito-borne Cache Valley orthobunyavirus (CVV) as well as the midge-borne Sathuperi (SATV) orthobunyaviruses after their ability to grow in Aag2 cells was verified (S4A Fig).

Similar to BUNV, silencing of Ago2 or Piwi4 promoted CVV replication, whereas Ago1 knockdown reduced CVV replication (Fig 3C). Moreover, CVV-infected, Piwi4-silenced Aag2 cells showed cytopathic effects not observed for any of the other knockdown experiments with this virus (S4B Fig). For midge-borne SATV, an increase in replication was observed in cells silenced for Ago2 and Ago1. However, no significant effect on SATV replication was observed in cells treated with Piwi4 dsRNA (Fig 3C). Successful silencing of transcripts by sequence specific dsRNAs was verified by quantitative RT-PCR (Figs 3D and 4C).

In short, Ago2 silencing led to a consistent increase in virus replication for mosquito and midge-borne orthobunyaviruses, supporting an antiviral activity of the exo-siRNA pathway. Similar effects were observed for Piwi4 silencing in the case of mosquito-borne viruses, but not midge-borne viruses.

Silencing of Ago1 resulted in a decrease of replication of the tested mosquito-borne viruses, suggesting an importance of the miRNA pathway for a successful infection in Aag2 cells for these viruses. In contrast, silencing of Ago1 resulted in an increase of SBV and SATV, albeit only slightly significant.

## Discussion

The RNAi response is a major antiviral response in arthropods against arbovirus infection. The activated exo-siRNA pathway plays a role in a variety of organisms, including mosquitoes and the model insect *D*. *melanogaster*. In contrast, the antiviral activity of the piRNA pathway and the production of viral specific piRNA molecules have been restricted to mosquitoes, especially *Aedes* spp. Besides, interactions between the miRNA pathway and viruses have been reported in several organisms [65, 66], acting either pro- or antiviral. This can be by expression changes of vector/host miRNAs or viral encoded miRNAs which can either directly target the virus or have host/vector targets, resulting in changes of the cell environment. Previous research has often used *D*. *melanogaster* to investigate the interaction between the insect RNAi response and different viruses including arboviruses; however little is known about the specificity of the antiviral response in vector- or non-vector arbovirus interactions. Knockdown experiments of key proteins of the exo-siRNA, miRNA and piRNA pathway in mosquito cells and orthobunyavirus infection, either mosquito- or midge-borne, supports the broad antiviral activity of the exo-siRNA pathway, based on an observed increase in virus replication upon Ago2 silencing for all viruses used. In contrast, the miRNA pathway seemed to be only important for virus replication in the case of a virus-vector match. miRNA expression is often species or even tissue specific and some miRNA-arbovirus interactions have been reported [67]. Whether a similar interaction is important for BUNV and CVV infection in mosquitoes has to be investigated in the future.

Infections with both mosquito-borne and midge-borne viruses were able to induce viral specific piRNAs in the used mosquito cell line. However, the antiviral activity of the piRNA pathway was only confirmed for the mosquito-borne viruses in the Piwi4 knockdown cells. Interestingly knockdown of other piRNA pathway members indicated that they may have pro-viral activities, knockdown of Piwi6 and Ago3 had replication suppressive effects on the mosquito-borne BUNV while knockdown of these proteins had no effect on the midge-borne SBV, whereas knockdown of Piwi5 reduced SBV replication but not BUNV replication. The meaning and biological relevance of these observations is not yet clear. Co-silencing/infection experiments and sequencing of virus-derived small RNAs from such cells may give clues to how different Piwi proteins interact, their role in piRNA-like small RNA production and potentially a hierarchy of protein effector and regulatory functions within this pathway. Perhaps, similar to other RNA binding proteins some of these proteins are important for promoting virus replication though this would require in depth analysis of RNA protein interactions and/or protein-protein interaction studies. Little is known about the piRNA pathway in mosquitoes, the production of virus specific piRNAs and how/if they target the virus. Recently, it has been shown that Piwi5 (and to a lesser extent Piwi6) and Ago3 are needed for virus-derived piRNA production [59, 60]; however, no antiviral activity has previously been linked to these transcripts. In contrast, Piwi4 seems not to be involved in virus-derived piRNA production [59]; however knockdown of Piwi4 can result in antiviral activity for mosquito-borne viruses, like SFV [47]. Similar antiviral activity of Piwi4 is observed for the mosquito-borne orthobunyaviruses: CVV and BUNV, but not for midge-borne orthobunyaviruses (SBV and SATV). This is especially striking as both the mosquito-borne BUNV as well as midge-borne SBV produce similar amounts of virus-specific piRNA molecules in infected Aag2 cells. This would suggest that the antiviral activity of Piwi4 is species specific and either acts as an effector protein downstream of the virus specific-piRNA production or through a different as yet unidentified pathway. Interestingly, no viral specific piRNAs have been reported in midges so far; although this has only been investigated in the *C*. *sonorensis*-derived KC cell line and not whole midges [40].

Interestingly, it has been shown that infection of mosquitoes and mosquito-derived cultured cells with arboviruses can lead to generation of virus derived cDNA forms, which are important for mosquito tolerance to virus infection and survival [68]. These DNA forms have the potential to become the template for small RNA production and could therefore determine the action of RNAi pathways on acute arbovirus infection. If the generation of cDNA forms in mosquitoes and derived cells is mosquito-borne virus specific (not occurring with e.g. midge borne viruses) is not known, but could explain the observed species specific antiviral activity of Piwi4.

Overall, these results show the importance to investigate the antiviral RNAi response in vector cells to understand the complex interaction between virus-vector interplay and its effect on tropism.

## Materials and Methods

### Cell lines

BSR-T7/5 cells [derived from the BSR clone of baby hamster kidney cells-21 [BHK-21] and stably expressing T7 RNA polymerase [69]; a kind gift of Dr. K.K. Conzelmann, Max-von-Pettenkofer Institut, Munich, Germany] were maintained in Glasgow minimal essential medium (GMEM) supplemented with 10% tryptose phosphate broth (TPB), 10% fetal calf serum (FCS), and 1 mg/mL geneticin. BHK-21 cells were maintained in GMEM supplemented with 10% TPB, 10% newborn calf serum (NCS) and 1% penicillin/streptomycin (P/S). Sheep choroid plexus cells (CPT-Tert) [70] were grown in Iscove's modified Dulbecco's media (IMDM) supplemented with 10% FCS and 1% P/S. Vero E6 cells were grown in Dulbecco’s modified Eagle’s medium (DMEM) and 10% FCS. BSR-T7/5, BHK-21, CPT-Tert and Vero E6 cells were grown at 37°C and 5% CO_2_. *Ae*. *aegypti*-derived Aag2 cells were maintained in L-15 medium supplemented with 10% TBP, 10% FCS and 1% P/S at 28°C.

### Plasmids

Plasmids to generate full-length BUNV antigenome RNA transcripts [pT7riboBUNL(+), pTVT7RBUNM(+), and pT7riboBUNS(+)] have been described previously [71, 72]. pTVT7RBUNM-NL was generated by PCR-directed internal deletion to replace the coding region of the NSm cytoplasmic tail (residues 395 to 456) with that of Nano luciferase (NL). A five amino acid linker, GASGA, was inserted between the NSm transmembrane domain (TMD) and the N-terminus of NL. To facilitate the cloning of NL, a unique *Kpn*I restriction enzyme site was introduced at nt 1419 in the BUNV M segment cDNA (S3A Fig). The plasmids, pUCSBVST7, pUCSBVMT7 and pUCSBVLT7, used to rescue SBV have been described previously [73]. pUCSBVT7-NL was generated through the introduction of two unique restriction sites, *MIu*I and *Xho*I, in the SBV M segment (provided by M. Varela; University of Glasgow). The restrictions sites were used to delete 90 nt of NSm (corresponding to nt 1235–1325 in JX853180.1) and NL was subsequently cloned in the deletion site. NL is a small luciferase subunit (19 kDa) from the deep sea shrimp *Oplophorus gracilirostris* with significantly increased luminescence expression and signal half-life as well as specific activity in mammalian cells compared to both *Firefly* and *Renilla* luciferases. NL uses a novel imidazopyrazinone substrate (furimazine) [74].

### Virus rescue by reverse genetics and plaque assay

Rescue experiments were performed as essentially described previously, with a modification [75]. Briefly, BSR-T7/5 cells were transfected with a mixture of plasmids comprising 0.5 μg each of pT7riboBUNL(+), pT7riboBUNS(+) and either TVT7RBUNM(+) or TVT7RBUNM-NLuc cDNA. At 4 hours p.t., 2 ml growth medium was added and incubation continued for 5–11 days at 33°C until cytopathic effect (CPE) was evident. Virus titre was determined by plaque assay on BSR-T7/5 cells. Cells were fixed in 4% formaldehyde and plaques stained in 0.01% toluidine blue. The rescue of SBV-NL was performed using pUCSBVST7, pUCSBVMT7-NL and pUCSBVLT7 as described for BUNV but with the exception that 1 μg of each plasmid was used and that the plaque assay was performed using BHK-21 cells.

### Preparation of virus working stocks

BUNV, BUNV-NL, SBV, SBV-NL, CVV and SATV stocks were grown in BHK-21 cells. Cells were infected with viruses at a multiplicity of infection (MOI) of 0.01 PFU/cell and incubated at 33°C. Virus-containing cell supernatant was harvested when CPE was evident (usually 2–4 days p.i.), cleared by centrifugation and stored at -80°C. Virus titres of BUNV and CVV were determined by plaque assay on BHK-21 cells and those of SBV and SATV on CPT-Tert cells.

### Metabolic radiolabelling and immunoprecipitation

Procedures for metabolic radiolabelling and immunoprecipitation of BUNV proteins were described previously [76]. Briefly, at 24 hours p.i., BSR-T7/5 cells were labelled with [^35^S]methionine (50 Ci) for 2 hours and then lysed, on ice, in 300 μl of non-denaturing RIPA buffer (50 mM Tris-HCl [pH7.4], 1% Triton X-100, 300 mM NaCl, 5 mM EDTA) containing a cocktail of protease inhibitors (Roche). For immunoprecipitation assays, BUNV viral proteins were immunoprecipitated with anti-BUNV antibody, a rabbit antisera raised against purified BUNV [77] that had been conjugated to magnetic Protein A-Dynabeads (Life Technologies). Viral proteins were analysed by SDS-PAGE under reducing conditions.

### Plaque assays in mammalian cells

The plaque phenotype of BUNV and BUNV-NL in BSR cells (routinely grown in GMEM supplemented with 10% foetal calf serum at 37°C in a 5% CO_2_ incubator) was investigated by plaque assay. Cells were seeded into 12-well plates at a density of 1.2 x 10^5^ cells/well and left to adhere overnight. Cells were infected with BUNV or BUNV-NL at a MOI of 0.05 and cells were fixed with 4% formaldehyde-PBS and stained with 0.1% crystal violet blue solution at 3 days post infection.

### Virus growth assays in mosquito cells

Growth of BUNV, SBV, CVV and SATV in Aag2 cells was assessed. Briefly, Aag2 cells were seeded into 24-well plates at a density of 1.7 x 10^5^ cells/well and left to adhere overnight. Cells were then infected with viruses at MOI 1 (BUNV, CVV) or MOI 0.01 (SBV, SATV) and culture supernatant was harvested at different time points p.i. Viral titres were determined by plaque assays on BHK-21 cells (BUNV, CVV) or CPT-Tert cells (SBV, SATV).

### Immunofluorescence assays

Aag2 cells were seeded into 24-well glass bottom plates at a density of 1.7 x 10^5^ cells/well and infected with BUNV, BUNV-NL, SBV or SBV-NL at a MOI of 0.01 for 48 hours. Cells were fixed and stained with anti-BUNV N or–SBV N antibody [78, 79]. Goat anti-rabbit Alexa Fluor 488 antibody (Molecular Probes) was used to detect primary antibodies and cells were mounted using Vectashield mounting media containing DAPI (Vectorlabs). Images were taken using the EVOS FL Cell Imaging System.

### Small RNA deep sequencing

Small RNA sequencing of BUNV-infected Aag2 and U4.4 cells was carried out by Edinburgh Genomics (University of Edinburgh) using the Illumina HighSeq 2000 platform, as previously described [40]. In short, 2.6 x 10^6^ Aag2 cells/well were seeded into a 6-well plate and left to adhere overnight. Cells were infected with BUNV at a MOI of 10. At 24 hours p.i., RNA was isolated from individual wells using 1 ml TRIzol (Life Technologies) followed by purification, sequencing, analysis and mapping of virus specific small RNAs using viRome [80]. Small RNA sequences were submitted to the European Nucleotide Archive (accession number PRJEB15203). Data for SBV are referenced under [40]. The actual number of reads for each experiment is shown S1 Table.

### Silencing of RNAi pathway components

Silencing of *Ae*. *aegypti* Ago2, Piwi4, and Ago1 was performed in Aag2 cells using dsRNAs as previously described [81]. dsRNA targeting eGFP was used as control. Silencing of transcripts was confirmed by qRT-PCR using Fast SYBR Green Master Mix (Applied Biosystems) and the corresponding primers [S2 Table; [81]]; on an ABI 7500 Fast real-time PCR instrument. *Ae*. *aegypti* S7 ribosomal transcript was used as housekeeping gene for relative quantification as previously described [81]. At 24 hours p.t., cells were infected with BUNV-NL, SBV-NL, CVV or SATV at a MOI of 0.01. At 48 hours p.i. cells were either lysed in passive lysis buffer and NL luminescence was measured (BUNV-NL and SBV-NL) or RNA was isolated using TRIzol (CVV and SATV), followed by cDNA production and qRT-PCR analysis. cDNA synthesis was performed using random hexamer primers (Promega) and SuperScript III reverse transcriptase (Life Technologies). qRT-PCR was performed using Fast SYBR Green Master Mix using corresponding primers (S2 Table). *Ae*. *aegypti* S7 ribosomal transcript was used as housekeeping gene.

## Supporting Information

S1 Fig SBV-specific small RNAs production in infected Aag2 and KC cells. Size distribution of SBV-specific small RNAs in *Ae*. *aegypti*-derived Aag2 cells at 48 hours p.i. **(A)** or *C*. *sonorensis* KC cells **(B)** at 24 hours p.i. The y-axis indicates read frequency; the x-axis indicates the length of the small RNAs (nt). Red indicates the small RNAs mapping to the antigenome and green the small RNAs mapping to the genome of L, M and S segments.
(TIF)

S2 Fig BUNV-specific small RNAs in infected U4.4 cells at 24 hours p.i. against L, M and S segment. **(A)** Size distribution of BUNV-specific small RNAs. The y axis indicates read frequency; the x axis indicates the length of the small RNAs (nt). Red indicates the small RNAs mapping to the antigenome and green the small RNAs mapping to the genome. **(B)** Relative nucleotide frequency and conservation per position of 24–30 nt small RNAs mapping to the genome or antigenome of BUNV S, M and L segments. Sequence is represented as DNA. **(C)** Frequency distribution of the 21 nt and 24–30 nt BUNV-specific small RNAs across the antigenome (positive numbers and red) from the 5` to 3` and genome (negative numbers and green) from the 3` to 5`.
(TIF)

S3 Fig Generation and characterization of recombinant BUNV or SBV expressing Nano luciferase (BUNV-NL or SBV-NL). **(A)** Construction of TVT7BUNM-NL in which the coding region of the NSm cytoplasmic tail (residues 395 to 455) was replaced by that of Nano luciferase (NL); BUN-NL GPC was cleaved into Gn, Gc, and NSm-NL chimeric protein. The fused NL is shown in orange, signal peptide (sp) in the grey box and transmembrane domain (TM) in the black box. The amino acid positions at the boundary of each protein are marked on top of Wt BUNV GPC. **(B)** Comparison of protein profiles of BUNV and BUNV-NL. BSR-T7/5 cells were infected with BUNV and BUNV-NL at MOI of 0.5 and labelled with [^35^S]methionine at 24 hours p.i for 20 hours. Viral proteins were precipitated with anti-BUNV antibody and analysed by 12.5% SDS-PAGE tris-glycine gel under reducing conditions. Positions of viral proteins are indicated. **(C)** Comparison of plaque phenotypes of BUNV and BUNV-NL on Vero E6 cells. Cells were fixed with 4% formaldehyde-PBS and stained with 0.1% crystal violet blue solution. **(D)** Immunofluorescence of Aag2 cells infected with either BUNV-NL or SBV-NL at MOI 0.01 at 48 hours p.i. Anti-BUNV or anti-SBV N primary antibody, followed by an anti-rabbit Alexa Fluor 488-conjugated secondary antibody (green) and nucleic acid staining with Dapi (blue) was used.
(TIF)

S4 Fig Growth of CVV, SBV and SATV in Aag2 cells. **(A)** Aag2 cells were infected with CVV (MOI 1), SBV, or SATV (MOI 0.01) and culture supernatants were harvested at different time points p.i. as indicated. Viral titres were determined by plaque assays on BHK-21 cells (CVV) or CPT-Tert cells (SBV, SATV). Graphs represent one experiment performed in triplicate. Error bars represent standard errors of the means (SE). **(B)** Aag2 cells were transfected with dsRNA either specifically to Piwi4 or eGFP as control, followed by CVV infection at 24 hours p.t. Images of cells shown were taken at 48 hours p.i using the EVOS FL Cell Imaging System.
(TIF)

S1 Table Actual deep sequencing reads for the data analysed in this study. The actual numbers of small RNA reads obtained in each experiment performed and analysed in this study are outlined in the table. The total number of reads against each virus segment as well as the number of reads of each size for each repeat is shown.
(XLSX)

S2 Table Primer sequences used in this study. (DOC)
