# Supplementary figures and images for: The Antiviral RNAi Response in Vector and Non-vector Cells against Orthobunyaviruses
Source: PLoS Negl Trop Dis. 2017 Jan 6;11(1):e0005272. doi: 10.1371/journal.pntd.0005272 (PMC5245901; doi:10.1371/journal.pntd.0005272)

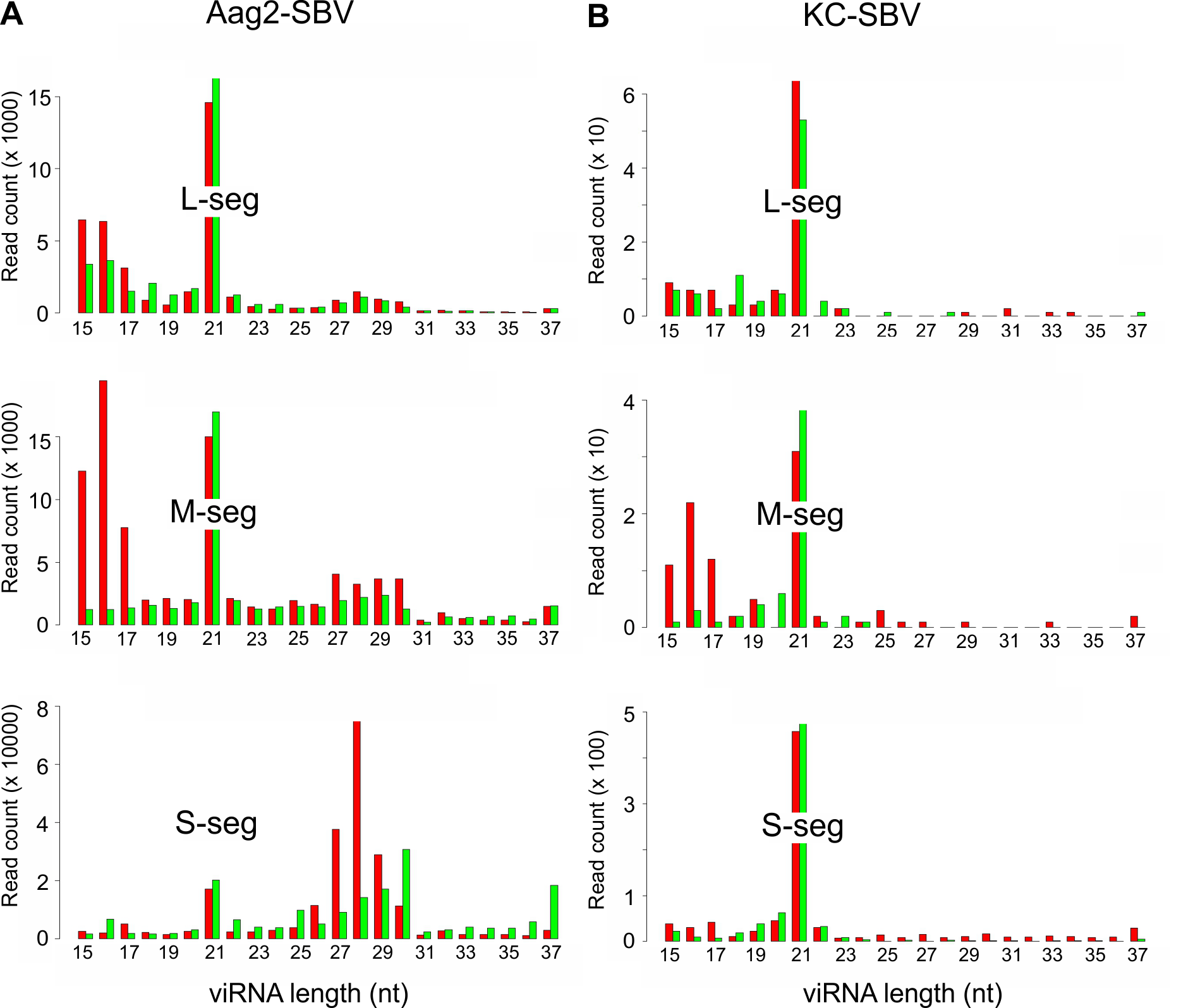

Supplement: S1 Fig — Size distribution of SBV-specific small RNAs in Ae. aegypti-derived Aag2 cells at 48 hours p.i. (A) or C. sonorensis KC cells (B) at 24 hours p.i. The y-axis indicates read frequency; the x-axis indicates the length of the small RNAs (nt). Red indicates the small RNAs mapping to the antigenome and green the small RNAs mapping to the genome of L, M and S segments. (TIF) [file pntd.0005272.s001.tif]

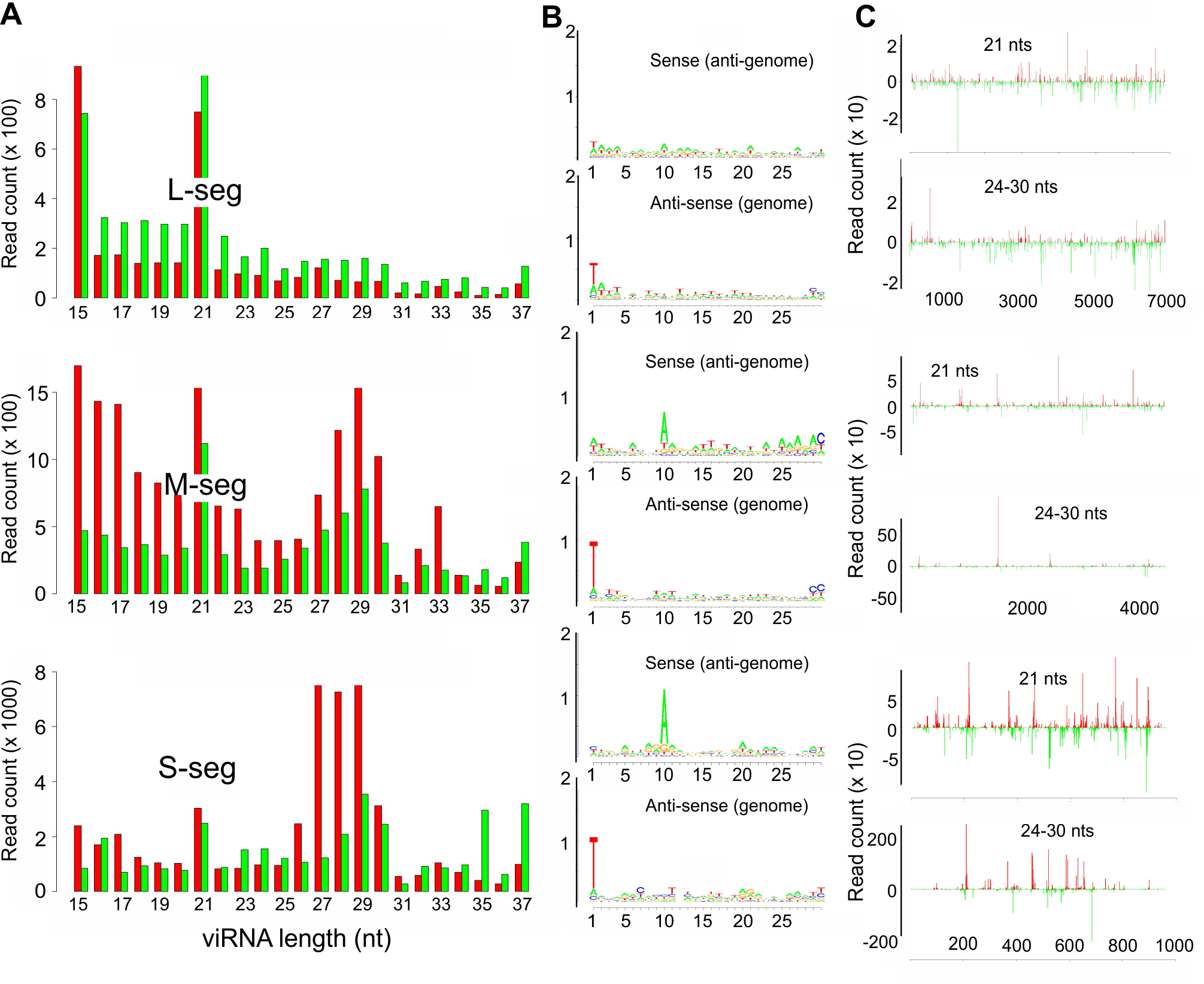

Supplement: S2 Fig — (A) Size distribution of BUNV-specific small RNAs. The y axis indicates read frequency; the x axis indicates the length of the small RNAs (nt). Red indicates the small RNAs mapping to the antigenome and green the small RNAs mapping to the genome. (B) Relative nucleotide frequency and conservation per position of 24–30 nt small RNAs mapping to the genome or antigenome of BUNV S, M and L segments. Sequence is represented as DNA. (C) Frequency distribution of the 21 nt and 24–30 nt BUNV-specific small RNAs across the antigenome (positive numbers and red) from the 5` to 3` and genome (negative numbers and green) from the 3` to 5`. (TIF) [file pntd.0005272.s002.tif]

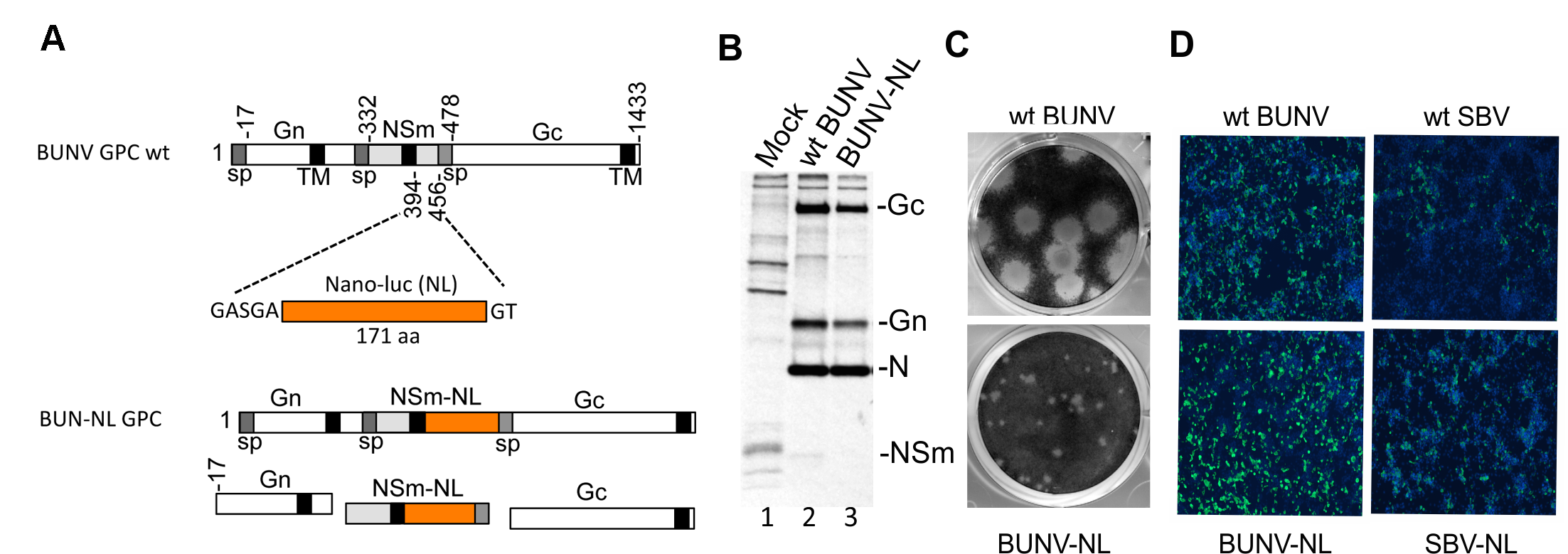

Supplement: S3 Fig — (A) Construction of TVT7BUNM-NL in which the coding region of the NSm cytoplasmic tail (residues 395 to 455) was replaced by that of Nano luciferase (NL); BUN-NL GPC was cleaved into Gn, Gc, and NSm-NL chimeric protein. The fused NL is shown in orange, signal peptide (sp) in the grey box and transmembrane domain (TM) in the black box. The amino acid positions at the boundary of each protein are marked on top of Wt BUNV GPC. (B) Comparison of protein profiles of BUNV and BUNV-NL. BSR-T7/5 cells were infected with BUNV and BUNV-NL at MOI of 0.5 and labelled with [35S]methionine at 24 hours p.i for 20 hours. Viral proteins were precipitated with anti-BUNV antibody and analysed by 12.5% SDS-PAGE tris-glycine gel under reducing conditions. Positions of viral proteins are indicated. (C) Comparison of plaque phenotypes of BUNV and BUNV-NL on Vero E6 cells. Cells were fixed with 4% formaldehyde-PBS and stained with 0.1% crystal violet blue solution. (D) Immunofluorescence of Aag2 cells infected with either BUNV-NL or SBV-NL at MOI 0.01 at 48 hours p.i. Anti-BUNV or anti-SBV N primary antibody, followed by an anti-rabbit Alexa Fluor 488-conjugated secondary antibody (green) and nucleic acid staining with Dapi (blue) was used. (TIF) [file pntd.0005272.s003.tif]

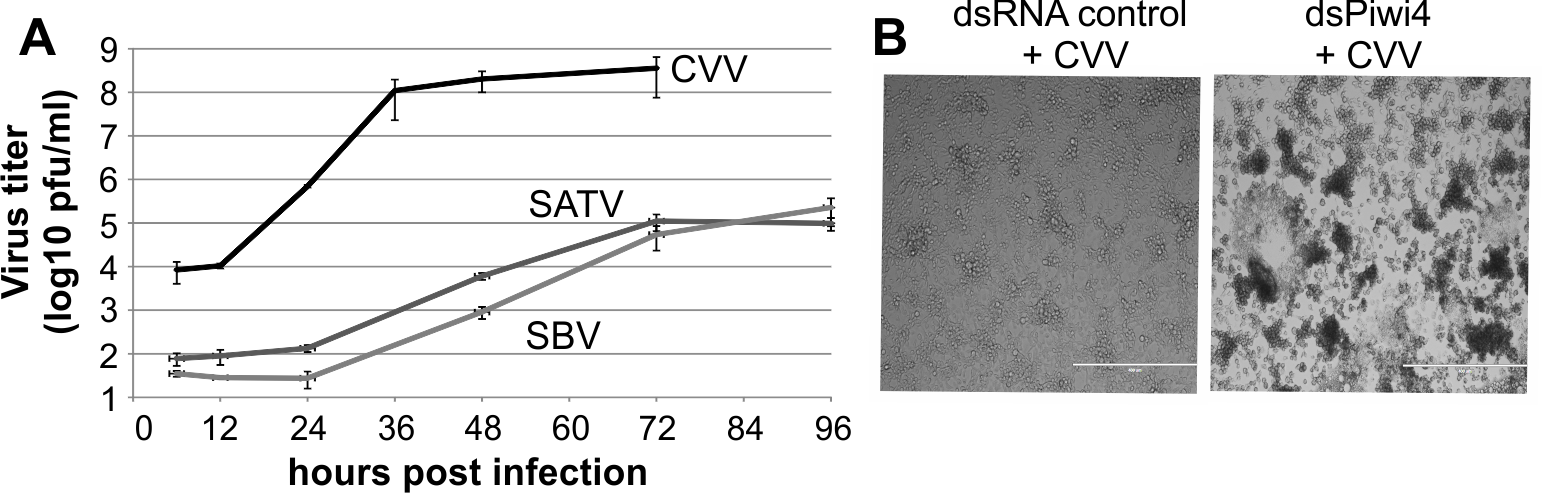

Supplement: S4 Fig — (A) Aag2 cells were infected with CVV (MOI 1), SBV, or SATV (MOI 0.01) and culture supernatants were harvested at different time points p.i. as indicated. Viral titres were determined by plaque assays on BHK-21 cells (CVV) or CPT-Tert cells (SBV, SATV). Graphs represent one experiment performed in triplicate. Error bars represent standard errors of the means (SE). (B) Aag2 cells were transfected with dsRNA either specifically to Piwi4 or eGFP as control, followed by CVV infection at 24 hours p.t. Images of cells shown were taken at 48 hours p.i using the EVOS FL Cell Imaging System. (TIF) [file pntd.0005272.s004.tif]
