# Supplementary material for: The Antiviral RNAi Response in Vector and Non-vector Cells against Orthobunyaviruses
Source: PLoS Negl Trop Dis. 2017 Jan 6;11(1):e0005272. doi: 10.1371/journal.pntd.0005272 (PMC5245901; doi:10.1371/journal.pntd.0005272)
Supplement: S2 Table — (DOC) [file pntd.0005272.s006.doc]

**S2 Table.** Oligonucleotides used in this study.

| Application | Primer name | Sequence (5`-3`) | Gene accession number |
| --- | --- | --- | --- |
| Silencing of *Ae. aegypti* Piwi5 transcripts using dsRNA | T7-Piwi5-F | gtaatacgactcactatagggcgtaatgttgctgtttcgaatg | gi|765339741 |
|  | T7-Piwi5-R | gtaatacgactcactataggggatttggaacaattagaggtg | gi|765339741 |
| Quantification of *Ae. aegypti* Piwi and Ago3  transcripts by  qRT-PCR | Aae-Piwi4-F | cttctccaccacagccaatg | gi|765335683 |
|  | Aae-Piwi4-R | gtccaatctgcctgttctcca | gi|765335683 |
|  | Aae-Piwi5-F | cagttttggaagacagagttgga | gi|765339741 |
|  | Aae-Piwi5-R | cctgccgtcactttgtaattttc | gi|765339741 |
|  | Aae-Piwi6-F | tccgacgttttcaagttttgga | gi|765339742 |
|  | Aae-Piwi6-R | cactttacactgatcctgctcg | gi|765339742 |
|  | Aae-Ago3-F | tgctccagacgacggttttg | gi|157117036 |
|  | Aae-Ago3-R | gggtcaatataacggctcccag | gi|157117036 |
| **Quantification of CVV and SATV RNA by qRT-PCR** | CVV-F | ccagacatagcacccaccatt | gi|5731965 |
|  | CVV-R | tcaccagcgaaatcccaatca | gi|5731965 |
|  | SATV-F | tgtccaactccaataccgcat | Reference 77 |
|  | SATV-R | ccccattcttcacaaccccaa | Reference 77 |
